# Detecting Emotional Evolution on Twitter during the COVID-19 Pandemic Using Text Analysis

**DOI:** 10.3390/ijerph18136981

**Published:** 2021-06-29

**Authors:** Javier Cabezas, Daniela Moctezuma, Alberto Fernández-Isabel, Isaac Martín de Diego

**Affiliations:** 1 Data Science Laboratory, Rey Juan Carlos University, 28933 Móstoles, Spain; alberto.fernandez.isabel@urjc.es (A.F.-I.); isaac.martin@urjc.es (I.M.d.D.); 2 Centro de Investigación en Ciencias de Información Geoespacial, Tlalpan 14240, Mexico; dmoctezuma@centrogeo.edu.mx

**Keywords:** sentiment analysis, COVID-19, Twitter, dynamic sentiment, social networks, pandemic evolution

## Abstract

Early in 2020, an unexpected and hazardous situation occurred threatening and challenging all of humankind. A new coronavirus called SARS-CoV-2 was first identified in Wuhan, China, and its related disease, called COVID-19, has induced one of the most dangerous crises at a global level since World War II. The ultra-fast transmission rate of the virus and the high mortality rate led the World Health Organization (WHO) to officially declare the situation a pandemic. Governments, for their part, were forced to implement unprecedented mobility restrictions and cease a large part of their economic activities. These facts triggered multiple reactions from people who expressed their feelings mainly through social networks (like Twitter), using them as vectors of information and opinion. In this paper, a study carried out in different Spanish speaking countries (Chile, Mexico, Peru, and Spain) is presented, which addresses the manner in which the evolution of the pandemic outbreak has affected the emotions expressed by individuals on Twitter over the last 13 months (from March 2020 to March 2021). We used a total of 3 million tweets to achieve this task. We made use of a well-known framework called *EmoWeb* to capture the dynamic variation in the sentimental value of pandemic-related words. The results reflect to what degree the pandemic and its derived problems have influenced and affected the population of the selected countries in different ways. The outcomes also illustrate the evolution over time of opinions published on Twitter regarding several topics related to COVID-19.

## 1. Introduction

Human beings have been historically defined by their ability to feel emotions [1]. These different emotions affect their behaviour and decision-making processes. Furthermore, these emotions are able to provoke changes in attitudes and behaviour as well as induce irrational acts [2]. This leads us to the study of human sentiments and the behaviour associated with them which has become a key issue of our time.

Delving into the behaviour of human beings, they can be classified as gregarious animals by definition [3], and they usually need social interactions with other members in the community in order to maintain their emotional stability [4]. This fact has made them prone to express emotions and communicate them to other individuals since prehistoric times. This procedure has led to developing complex societies able to collaborate and evolve to successfully pursue common goals [5]. As a consequence of these interactions, reinforcement situations could be produced according to a specific topic which would generate a general or collective sentiment in the community.

The study of this general sentiment in a modern society constitutes an important vector to address its psychological affectation regarding different past events [6]. On the other hand, nowadays, social networks have become the most common source of information used by humankind to express sentiments and opinions. Thus, the study of social networks has emerged as a key means of evaluating the mental health, opinions, and the general sentiment of the population with regard to any specific event [7]. Moreover, social media can also be used to detect the trends and evolution of such events by evaluating the most common expressions (e.g., protests or complaints) and specific words (e.g., occurrence of words in similar texts) used b individuals when they manifest their sentiments or opinions [8].

Currently, the most relevant event is the appearance of the new coronavirus named SARS-CoV-2, first identified in Wuhan, China, in early 2020 [9]. This new pathogen is associated with a respiratory infectious disease called COVID-19 which has induced one of the most dangerous crises at a global level since World War II. The ultra-fast transmission rate of the virus [10] is capable of leading to the collapse of hospitals and health centres with thousands of infected patients [11]. Though the mortality rate of the virus is significantly low, there have been millions of registered deaths all over the world. For these reasons, the World Health Organisation (WHO) officially declared the situation on 11 March 2020.

This pandemic outbreak forced the implementation of a range of measures through the official statements by governments of most countries all over the world. Among these measures, unprecedented mobility restrictions and mandatory isolation requests were implemented. The populations of most countries were directly confined to their homes, and businesses were (almost in their totality) closed; thus, the economy of these countries came to a harsh standstill [12,13]. This situation was aggravated with mandatory quarantine periods for infected individuals who suffered the disease in a mild way, or who had been in close contact with infected people [14].

At the social level, the social distance between individuals and the avoidance of almost any kind of physical contact were among the most relevant implemented measures. This produced modifications in the behaviour of humans. Moreover, the use of medical face masks was recommended and even mandatory in most countries. This also modified the way people behaved in close quarters, diminishing their ability to properly communicate by making gestures and facial expressions [15].

All these considerations have led to producing frustration among the population as well as mental and psychological affectation. Thus, people have used the Internet as a way of escape, increasing virtual communication to compensate for the decrease and overall worsening of human interactions at the social level. In particular, social networks have become a main vector of expression, where individuals are capable of sharing their opinions and discomfort with the pandemic situation, whilst also searching for information about symptoms, vaccination procedures, and any kind of news related to COVID-19 [16].

For these reasons, a comprehensive study of well-known social networks can provide valuable information regarding the feelings of the individuals during the pandemic outbreak. Thus, this study comprised four countries where Spanish is the official language, namely Chile, Mexico, Peru, and Spain. The social network evaluated was Twitter [17], because it is one of the most used in the world and its textual content and the emoticons provided its users are relatively affordable to process.

The *EmoWeb* framework [18] was selected to evaluate of sentiments expressed by individuals on Twitter. *EmoWeb* is a visual system which is able to dynamically estimate the evolution of sentimental value and to analyse the occurrence of words. It makes use of a lexicon as a seed to establish the initial sentiment polarity of predefined words. Then, the system captures the sentiment from the evaluated texts, learning new concepts and upgrading its knowledge. We note that the system was developed to analyse texts provided by the digital versions of well-known newspapers. Thus, it has to be adapted to study the textual content of social networks.

Some experiments were conducted for each of the countries of interest. They allowed illustrating the viability of the study consisting of a 5D word cloud provided by *EmoWeb* which dynamically evolves and trends plots that show the evolution of the sentimental values of specific keywords. These keywords are related to the COVID-19 outbreak, and they have been manually selected according to different relevant perspectives (e.g., economic or health issues).

The rest of the paper is organised as follows. Section 2 provides the foundations of the proposed study. Section 3 details how the information was recovered from Twitter and the processing steps that followed to generate the dynamic sentimental knowledge. Section 4 addresses the different developed experiments to confirm the quality of the study. Finally, Section 5 concludes and provides some future guidelines about the topic.

## 2. Background

This section provides the context of the study by addressing its scope and foundations. Thus, Section 2.1 introduces the COVID-19 pandemic outbreak and details its evolution in the countries of interest by focusing on the different produced waves. Section 2.2 discusses the different approaches related to sentiment analysis, highlighting the different perspectives at lexical and semantic levels of the language. Moreover, it also includes some details about the trends and the state of the art in the domain of application.

### 2.1. The COVID-19 Pandemic

In Wuhan, China, in early 2020, a new coronavirus was detected and named SARS-CoV-2 [9]. It causes a respiratory and vascular infectious disease called COVID-19. The main characteristic of this new pathogen is its capability to be transmitted among individuals sharing a reduced space through the respiratory tract. Though the mortality rate of the virus is significantly low, millions of deaths have been registered all over the world. As a consequence, COVID-19 was officially declared as a pandemic by the WHO on 11 March 2020 [19].

One year later, the outbreak remains ongoing and the number of confirmed cases in the world is continuously increasing. Today, there have been more than 160 million confirmed cases and more than 3 million deaths. This situation has had different effects on various countries, some of which only suffered the initial chaotic conditions, whilst others presented pronounced waves of contagion. Furthermore, some countries have suffered different numbers of waves of contagion (three or more, as there are even active waves at the moment), while others have only detected one or two waves [20]. This variation is a consequence of the characteristics of the population of each country, its level of industrial and technological development, and its vaccination capability. For instance, in Latin America, the outbreak came two or three weeks after it did Europe.

This situation has been reflected in social networks and information media [21,22]. Therefore, the study of these information vectors can determine how all these variations and the measures implemented by the different countries have affected the population. In this regard, the Oxford COVID-19 Government Response Tracker (OxCGRT) dataset [23] has analysed more than 180 countries since January 1st, 2020, to provide 20 indicators showing the responses of the different governments to the pandemic. These indicators tackle individual policies related to confinement and closure and concerning the economic and health systems. These indicators are likewise focused on four global indexes (government response; containment and health; stringency; and economic support) which in turn are very practical to both gather an overall impression of government activities and further proceed with cross-country comparative exercises. A similar study was performed in the Response2covid19 dataset [24], where two global indexes (economic intervention and rigidity of public health measures) were developed from a list of policy indicators for more than 200 countries covering the period from 1 January 2020 to 1 October 2020. The values for the designed global indexes range from 0 (no intervention) to 1 (all interventions are strict). Finally, it is also worth mentioning that the CoronaNet COVID-19 Government Response Event dataset [25] has analysed more than 12,000 policies taken by governments across the world since 31st December 2020 to assess their effectiveness. Furthermore, the scientific community is focused on providing new perspectives and research about the problem in order to advance its mitigation [26]. Psychological and sociological analysis were also included in these approaches, which can provide important assets in detecting the impact of the pandemic among the citizens of the world [27,28].

In this paper, the general sentiment generated during the evolution of the pandemic event was analysed through the information provided and the general sentiment of the Spanish-speaking population on social media. Thus, some countries in Latin America (Chile, Mexico, and Peru) and Europe (Spain) were selected for this purpose. The following sections describe some related works and general insights about each country.

#### 2.1.1. Chile

In Chile, the first confirmed case was registered on the 3 March 2020, and the first lockdown was established during the final week of March in seven municipalities of the Metropolitan Region. Currently, Chile reports more than 25,000 deaths, and more than 1.17 million infections.

It is important to indicate that when the pandemic began in Chile, the country experienced several dramatic events related to massive protests in October 2019, from which social gaps and damages ensued across the country which probably aggravated the situation.

**Figure 1:**
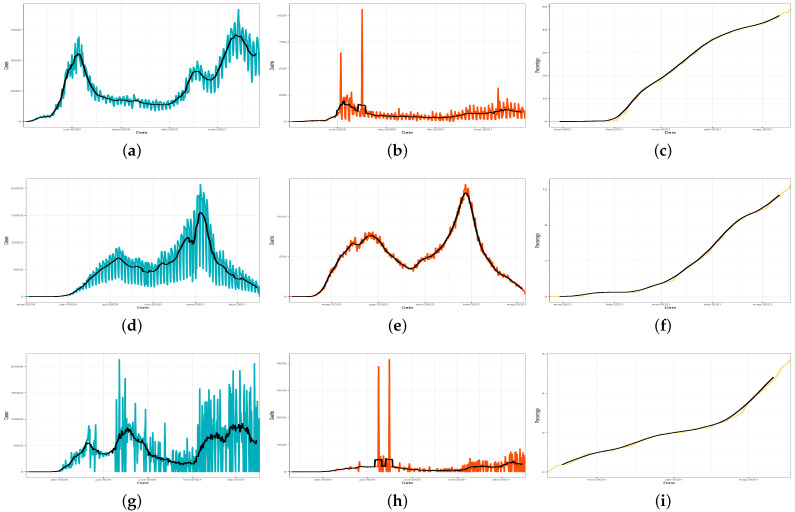

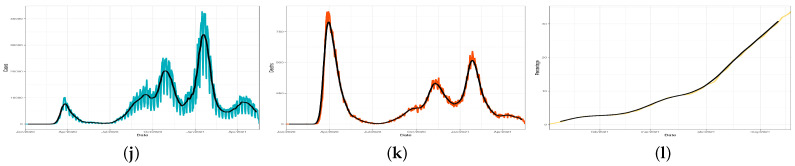
Confirmed cases, deaths, and vaccinated people per hundred individuals: (**a**) Chile cases; (**b**) Chile deaths; (**c**) Chile vaccines; (**d**) Mexico cases; (**e**) Mexico deaths; (**f**) Mexico vaccines; (**g**) Peru cases; (**h**) Peru deaths; (**i**) Peru vaccines; (**j**) Spain cases; (**k**) Spain deaths; and (**l**) Spain vaccines.

On the other hand, Chile is one of the countries in Latin America with the highest testing rates, in addition to a low mortality rate [29]. Figure 1a–c show the number of cases, deaths, and vaccines over time in Chile, respectively. Specifically, in Figure 1c, we can see that Chile also achieved a fast vaccines rate. In contrast, in Figure 1a, it can be observed that the number of infections is also increasing. This fact apparently shows a third wave of greater size than the previous ones. Likewise, the number of deaths (despite being collected with errors) shows an increment in the most recent months (see Figure 1b).

Both the studies performed in [23,24] (OxCGRT dataset) present indicators addressing the number of confirmed cases and confirmed deaths for the selected countries. A detailed analysis of both indicators allows us to corroborate the data previously presented in the respective figures for Chile. In addition, the related data corresponding to the rest of the countries are also validated.

With regard to vaccination, the OxCGRT dataset introduces a vaccination policy indicator (numerical values between [0,5]) which enhances the related discussions and is useful to both contextualise vaccine-related figures and proceed with several cross-country comparisons.

Table 1 and Table 2 summarise the information provided by the OxCGRT dataset with respect to the vaccination policy chosen by the selected countries. Table 1 details the target groups for the vaccination are depending on the policy value. With regard to Table 2, it illustrates the specific dates on which each country started to apply the corresponding policy value.

In this respect, Table 2 showed a very quick progression with a nearly monthly change in the policy value for Chile. This fact is also reflected in Figure 1c, where a pronounced slope revealed that almost 50% of the population is already vaccinated and that a potential upcoming change to the value (5) of the policy is likely.

Regarding the different studies about the country, some approaches have been published about several aspects of the COVID-19 outbreak and its effects. Examples of these are the health, education, employment, economic, psychological, and social aspects. All of these were analysed at different levels, such as for a specific country, community, or the whole world. For instance, at the psychological level, the question concerning the way in which the COVID-19 pandemic has affected humans and their behaviour is one of the most important to solve. It is a fact that depressive disorders among adolescents and adults are increasing, in addition to alcohol abuse [29]. Other studies, on the contrary, proceeded with a more general scope. For example, some of these focused on analysing the effect of lockdowns on the reduction in new cases. These effects are usually analysed in terms of high-income and low-income areas, detecting that small-area lockdown measures had a differential effect on high and lower-income populations [30]. This fact leads to thinking that mobility reduction measures are not equally effective in all types of areas in Chile.

#### 2.1.2. Mexico

In Mexico, the first confirmed case was reported on 27 February 2020. Today, Mexico reports more than 2.33 million infections, and more than 215,000 deaths.

The outbreak evolution in Mexico presents several waves over time. Nevertheless, contagions and deaths have considerably reduced by the time of writing. Figure 1d–f illustrate the number of cases, deaths, and vaccinations over time, respectively.

In terms of the vaccination policy, Table 2 illustrates its current status. The reduced efficiency and overall development of the Mexican public health system led to much slower vaccination progress, as demonstrated in Figure 1f, with approximately 12% of the population currently vaccinated.

Regarding studies about the pandemic in Mexico, the most relevant are those related to the opinion and impact of COVID-19 on Mexican citizens. This perspective is very generic and encompasses a great variety of impacted life dimensions such as healthcare, psychological and sociological aspects, economic, and environmental issues. It is common that Mexican citizens suffer from chronic diseases as hypertension, diabetes, and obesity, which has intensified the impact of the virus. Moreover, the economic impact has been huge because all non-essential activities were suspended. A global analysis of these issues can be found in [31].

In the case of text mining approaches similar to the study presented here, there are some relevant ones. These usually include a social media analysis where the textual information provided by individuals in Mexico is processed. Twitter and other social networks are common sources of this information [32]. These approaches report important conclusions about the behaviour of individuals, where the first perceivable sufficiently active mentions of the COVID-19 topic on social networks mainly occurred between 13 March 2020 and 20 March 2020. The main words associated with the keyword COVID-19 were *coronavirus*, *government*, and *contagious*.

#### 2.1.3. Peru

In Peru, the first confirmed case was registered on 6 March 2020. Currently, Peru reports more than 59,000 deaths and more than 1.76 million infections. According to the official Peruvian data, the numbers of cases, deaths, and vaccines are currently high. This fact seems slightly contradictory due to other countries in the world having experienced a significant improvement in all areas, or at least a noticeable reduction in the number of deaths as a consequence of the vaccination. Nevertheless, the number of vaccinations in Peru is growing exponentially (see Figure 1g–i).

The vaccination policy status indicated in Table 2 for Peru matches those observed in Mexico and Spain. However, it is pertinent to note that the Peruvian public health system is even less developed than the Mexican one. This fact is manifested by the data shown in Figure 1i, revealing that only 6% of the population is vaccinated. In addition, considering population size in Mexico (128 million) in comparison with the one in Peru (33 million), it seems reasonable to conclude that Peru represents the country with the worst performance in terms of vaccination process among the selected countries.

Regarding the published works related to the COVID-19 outbreak in the country, several works about Peruvian society were considered. For instance, the impact of the lockdown policy on homicide, suicide, and road traffic-related deaths are interesting aspects to evaluate [33]. The conclusion was the expected one, with a significant reduction in road traffic accidents due to the diminishing flow of vehicles and pedestrians. However, it is important to remark that the number of homicide and suicide deaths was not significantly reduced. These facts lead to analysing the psychological aspect of Peruvian society [34]. In a similar way to the proposal presented herein, Facebook and Twitter textual content was analysed to evaluate the stress, depression, and frustration levels of the citizens during the lockdown.

#### 2.1.4. Spain

In Spain, the first confirmed case was reported on 31 January 2020. Today, Spain officially reports more than 3.50 million cases, and more than 77,000 deaths. Thus, Spain is one of the countries most strongly affected by the COVID-19 pandemic. The social and economic impacts are without precedent.

The Spanish government implemented a series of social distancing and mobility restriction measures on 14 March 2020. However, at the end of March 2020, the number of deaths in Spain exceeded that of mainland China, and was only exceeded by that in Italy.

Regarding the studies analysing the impact of the pandemic, most of have been focused on people’s healths (see, for instance, [35,36]). In [37], it was shown that positive attitudes decreased gradually over time, reaching their lowest level during the week the lockdown was established, while negative attitude remained stable, without an increase during this period.

Other approaches have considered the impact of COVID-19 in Spain in terms of morbidity, mortality, and recovery [38]. In Spain, at least three waves of COVID-19 cases have been detected (see Figure 1j). The third wave was the one with the highest number of cases. However, Figure 1k shows that the first wave was the most severe in terms of mortality rate. With reference to the vaccines, it is important to note that the significant performance in vaccination results shown in Chile poses an important contrast with those presented by the other countries, especially Spain. This specific case draws a lot of attention due to the fact that the Spanish public health system is highly ranked, in rankings such as those of the WHO [39] and Bloomberg [40]. As illustrated in Table 2, Spain rapidly jumped to the second phase of its vaccination policy but did not advance further until five months later. Despite having the proper facilities and necessary provision of vaccines (under agreement with the European Commission), flawed political decisions and complex territorial organisation have led to a noticeable delay in the process, with only 30% of the population vaccinated to date, as shown in Figure 1l.

As evidenced by all the previous analyses, the four countries present similarities and differences in the context of the COVID-19 pandemic. The availability of official information also provides a solid reference against which to compare the conclusions reached from the present study based on Twitter data.

### 2.2. Sentiment Analysis

Sentiment analysis is a ramification in the natural language processing (NLP) domain [41]. It focuses on detecting, processing, and analysing the feelings and emotions expressed by human beings in their language (i.e., the natural language). These can be classified according to six basic perspectives: anger, fear, joy, repulsion, sadness, and surprise [42]. This classification is interesting for organising human facial expressions, but it presents some difficulties when it is used for textual content. These difficulties are mainly due to the fact texts usually have more than one of these perspectives, generating an overlap which sometimes produces a non-deterministic result.

Basic polarity approaches (i.e., negative, positive, and neutral polarities) appear as a solution to mitigate the problem in the domain of the textual information. This classification allows including a numeric scale (discrete or continuous) in order to measure the intensity of the polarisation [43].

The kind of systems that could address the calculation of this polarity can be classified according to the level of language at which they act [44]. On the one hand, at the lexical level (i.e., words are considered individually), the most common approaches are those based on a dictionary—usually named lexicon. Examples of these perspectives are SenticNet [45] and SentiWordNet [46]. On the other hand, at the semantic level (i.e., words are considered as a collective), the most common approaches are those based on statistics and machine learning (ML) models.

Statistical approaches [47] obtain the polarity of words focusing on the frequency of their appearance in several texts (usually a corpus of documents). Thus, words that appear close to other words within the previously defined polarity, frequently take analogous polarity values. This procedure allows increasing the knowledge of the system with new words with initial unknown sentimental polarity. This fact is the basis of the distributed semantic which is used by most of the ML approaches to learn possible patterns from text.

ML approaches are usually focused on predicting the polarity of complete sentences, paragraphs, or even whole documents [48]. Notice that they are not adequate to predict the sentiment polarity of individual words. Typically, there are supervised models that work through training and test sets.

Deep learning approaches are a particular type of ML approaches. They are among the most common ML models used to predict sentiment polarity in texts [49]. Furthermore, the rise of bidirectional encoders and transformers has meant an improvement in this domain. Thus, bidirectional encoders such as Representations from Transformers (BERT) [50] and other similar proposals process the text in both directions, as these are able to obtain more information from the context at the semantic level, and therefore better patterns in the text.

It is interesting to mention the apparition of hybrid perspectives where the lexical and semantic levels are mixed [51]. These systems usually comprehend a lexicon (i.e., a dictionary with several well-known lemmatised words and their related sentiment polarity) and some ML models [52]. Thus, these try to match words with the dictionary to find the predefined polarity. If none of the words of the sentence are matched, then the corresponding ML model predicts its sentiment polarity value.

Notice that all these perspectives consider the sentiment polarity as a constant value. However, the opinions and emotions of humans reflected in words can change over time [53]. Therefore, it is necessary to develop systems that address this issue. A well-known instance of these proposals is *EmoWeb* [18], which dynamically calculates the sentiment value of words using a well-known lexicon as the seed. For this purpose, an adapted version of *EmoWeb* was used in this paper to evaluate the general sentiment evolution in individuals during the COVID-19 outbreak using a whole year of tweets related to it.

## 3. Information Gathering and Processing

The present study consists of two stages which are executed in a sequential manner. The initial phase comprises both the gathering of raw data (tweets) from the selected source of information (Twitter) and the subsequent application of proper filtering processes according to predefined sets of rules given by keywords. The first set of rules restricts the data to the context of the COVID-19 pandemic (keywords such as *covid*, *covid19*, and *coronavirus*), producing a total of 9 million tweets during the period considered (March 2020 to March 2021). Subsequently, the second set of rules allows classifying the resulting tweets into five predefined topics of interest (*government*; *health*; *economy*; *employment*; and *vaccines*). This process reduces the size of the data to 3 million tweets. To conclude, there is a final set of rules targeting the consideration of the country information, which finally produces a set containing 105,497 tweets. The Section 3.1 provides further details on the manner in which this information is distributed among Chile, Mexico, Peru, and Spain, as well as the keywords utilised to cope with each topic.

Once the textual content of interest is available, the second phase is triggered when the aforementioned adapted version of the *EmoWeb* framework processes it. This system is able to estimate the sentiment polarity of words and its evolution over time. Section 3.2 introduces the framework and its most relevant functionalities.

### 3.1. Twitter Database Related to COVID-19

The resulting efforts from the filtering processes are summarised in Table 3, illustrating the number of tweets per topic and country to be processed by *EmoWeb* during the next stage. It is relevant to note that the outcomes shown in Table 3 demand appropriate prior tweet hydration processes to effectively infer the country information (in our case, Chile, Mexico, Peru, and Spain).

The topics were selected with the aim of addressing the leading concerns of the population in terms of COVID-19 and the prospects of a short-term foreseeable future in which the world has overcome the pandemic. In this regard, for topic 1 (*government*), we focused on collecting opinions on the measures and corrective actions imposed by the governments of the selected countries. In this scenario, *corruption* (“*corrupción*”), *management* (“*gestión*”), *politician* (“*político*”), and *democracy* (“*democracia*”) represent some of the main driving keywords utilised as filtering rules to gather the respective tweets. As for topic 2 (*health*), attention is drawn to capturing viewpoints relative to the psychological impact of the pandemic and the consequences of COVID-19 themselves. Keywords such as *depression* (“*depresión*”), *stress* (“*estrés*”), *psycologist* (“*psicólogo*”), and *anguish* (“*angustia*”) are among the representative filtering candidates applied. With reference to topic 3 (*economy*), due care is taken to compile relevant voices mainly discussing national macroeconomic indicators and mid-term recovery strategies for companies and local businesses. Keywords such as *business* (“*negocio*”), *company* (“*company*”), *debt* (“*deuda*”), *bankruptcy* (“*quiebra*”) and *GDP* (gross domestic product, “*PIB*”) constitute the major part of the debates observed in the different countries and therefore, the filtering set. Topic 4 (*employment*), for its part, addresses the conversations covering the abrupt changes that occurred in the working philosophy (e.g., working from home), and moreover, the staggering increment in unemployment rates in the countries. Keywords such as *Zoom*, *Skype*, *videocall* (“*videoconferencia*”), *unemployment* (“*desempleo*”) and *dismissal* (“*despido*”) constitute some of the filtering resources used in this context. Lastly, topic 5 (*vaccines*) tackles the perception of the prevention methods available and the explicitly manifested state of uncertainty with regard to the level of trust in them. Keywords such as *Astrazeneca*, *Pfizer*, *PCR*, *antigens* (“*antígenos*”) and *effectiveness* (“*eficacia*”) depict some of the most notorious filtering instances in this respect.

**Figure 2:**
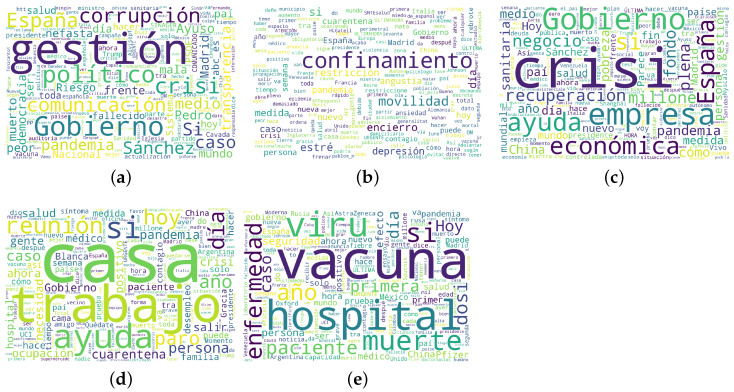
Word clouds per topic: (**a**) government; (**b**) health; (**c**) economic; (**d**) employment; (**e**) vaccines.

To conclude, Figure 2 exhibits one word cloud per topic. In this type of visualisations, the frequency of a word is defined by its size (the larger, the more frequent). To this end, the aforementioned set containing 3 million tweets was used. In addition, some COVID-19-related words were considered as stopwords to ensure the better visualisation of the stemming procedure applied.

### 3.2. The EmoWeb Framework

The present study bases its foundations on an adaptation of a former framework called *EmoWeb* [18]. This revised version aims to grant the original framework the ability to process the data streams originating on Twitter. Taking a general purpose lexicon created from the Spanish version of *SenticNet* [45] as a starting basis, the framework shows proficiency in applying text analysis techniques in conjunction with an unsupervised learning algorithm to effectively process incoming tweet datasets and extract relevant words that adhere to its lexicon, along with computed sentiment values.

The overall process is orchestrated by the use of time as a considered magnitude, which contextualises the knowledge acquired at a word level and confers the possibility of making the computed sentiments evolve. Consequently, word sentiments present a dynamic nature in which the strengthening or weakening calculations are applied according to the influence they are exerting or, in other words, following the trends detected in the input data. The framework similarly offers several visualisations for the outcomes arising from the aforementioned analysis, since similarity relationships between lexicon words through 5D word clouds and word sentiment fluctuation graphs over time are the most relevant.

**Figure 3:**
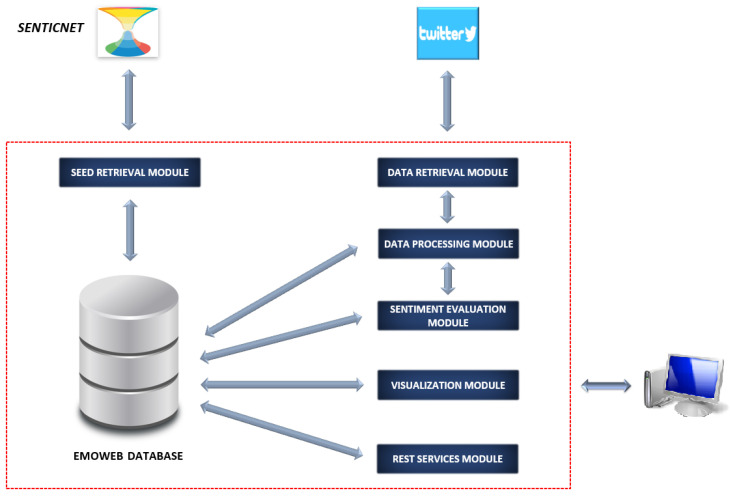
The *EmoWeb* framework architecture.

Figure 3 illustrates the architecture of the adaptation of *EmoWeb* and its major internal components. The *Seed Retrieval Module* takes responsibility for incorporating the commencing seed to the lexicon. This initial seed was given by the Spanish version of *SenticNet*, which offers 29,630 Spanish words along with their associated numerical values in the range of [−1,1], representing the sentiment polarities. This starting lexicon is enhanced with the new words learnt during the processing of the Twitter data.

Subsequent to this initial one-off phase, the *Data Retrieval Module* collects an input set of tweets created on a particular calendar day *d* and harvests relevant and necessary information (i.e., tweet hydration) as a mandatory step prior to triggering a request for the activities concerning the *Data Processing Module*.

The *Data Retrieval Module*, for its part, conducts tweet preprocessing activities including the removal of non-relevant information (considered in this work), such as smileys, emojis, special characters, mentions and hashtags, among others. Subsequently, the cleaned tweets in addition to some metadata information (tweet ID, creation date, etc.) are stored in the internal database. Subsequently, the *Data Processing Module* focuses on applying NLP activities on texts such as tokenisation, lemmatisation, and part of speech (PoS) tagging methods. The removal of the Spanish-related stopwords is also covered by the exercise.

As a result, the input tweet dataset is fully processed and sentiment scores are calculated for every belonging tweet. In addition, a well-nourished list of detected words is obtained. These words are properly stored in the lexicon in case they were not present in it previously.

Following the aforementioned actions, the endeavours encompassed by the *Sentiment Evaluation Module* are initiated. This module is accountable for initially computing (or updating) the sentiment values of all the words stored in the lexicon according to the trends detected. For each word, these required calculations were based on the sentiment score of the tweets where the word was detected during the calendar day *d* under analysis and the former sentiment value stored in the lexicon for the same word under scope.

The module was also liable for the management of an internal archive that stores the historical information on the sentiment values computed for the lexicon words. This archive plays a critical role in the duties performed by the *Visualisation Module*.

As stated before, the *Visualisation Module* produces relationships between lexicon words through 5D word clouds and word–sentiment fluctuation graphs over time. These visualisations conform to the main focus of the present paper where *EmoWeb* is exposed to tweets related to the COVID-19 pandemic and a graphical analysis is performed to establish a comparison between the different countries of interest.

## 4. Experiments

In this section, different experiments are presented to show the emotional evolution during the COVID-19 pandemic in the analysed countries regarding the previously described topics. As stated previously, a total of 9 million tweets were retrieved in a first set containing tweets related to COVID-19. Subsequently, from this set and according to the filtering keywords applied to make a selection for each topic, a subset of more than 3 million tweets was created. Finally, additional filtering was applied to only consider those tweets with their country information available (official information reported by Twitter), giving a total of 105,497 tweets.

**Figure 4:**
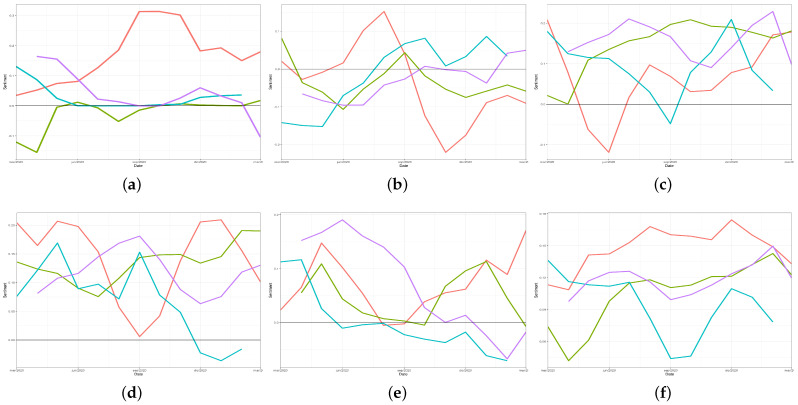
Sentiment evolution of words related to different topics per country: Chile in red, Mexico in green, Peru in blue, and Spain in purple: (**a**) democracy; (**b**) depression; (**c**) recovery; (**d**) meeting; (**e**) face mask; and (**f**) vaccine.

In order to compare the emotional evolution from the beginning of the pandemic in the different countries, Figure 4 shows the classical linear graph for sentiment values’ representation for a set of words. Analysing these figures, a negative trend can be noted regarding the word *democracy* (“*democracia*”) (see Figure 4a), probably as a result of the social problems associated with the dramatic reduction in the fundamental rights caused by the successive restrictions imposed by the government.

In Figure 4b,c, the evolution of the sentiment associated with the words *depression* and *recovery* (“*depresión*” and “*recuperación*”), could be related to the number of cases and deaths in each country.

In general, the word *meeting* (“*reunión*”) was always associated with a positive sentiment (Figure 4d). Regarding the expression *face mask* (“*mascarilla*” or “*cubrebocas*”), a negative trend presented in Spain and Peru, probably associated with the population’s fatigue regarding the need for its use. Figure 4f shows positive emotions related to the Spanish word for *vaccine* (“*vacuna*”) in all the considered countries. In Mexico and Spain (and to a lesser extent, Chile), there is a growing positive trend that could be associated with an increase in the number of people vaccinated.

The previous visualisation helps us understand the dynamic behaviour of the sentiments associated with some words over time, however, it is not able to extract potential relationships between them. To attend this point, word cloud visualisations were used. In these graphs, the colour of each word is related to the polarity of its sentiment. In this way, red is associated with a negative emotion and green with a positive one. The size of each word is associated with the frequency of its occurrence, being larger the more the word appeared in tweets. Finally, the distance between words represents their relationship, with smaller one being more related to one another.

**Figure 5:**
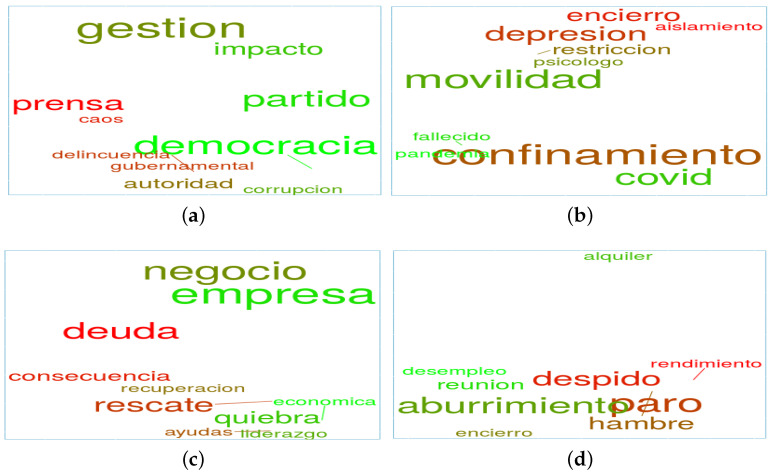
Relationships among words for different topics and countries. (**a**) government topic, in Spain; (**b**) health topic, in Peru; (**c**) economy topic, in Mexico; and (**d**) employment topic, in Chile.

Figure 5 presents the relationships between a set of words per topic (*government*; *health*; *economy* and *employment*) in different countries measured during March 2021.

For instance, regarding *government* topic, the relationship between the following words is analysed in Figure 5a for Spain: *authority* (“*autoridad*”); *chaos* (“*caos*”); *corruption* (“*corrupción*”); *democracy* (“*democracia*”); *delinquency* (“*delincuencia*”); *management* (“*gestión*”); *governmental* (“*gubernamental*”); *impact* (“*impacto*”); *political party* (“*partido*”); and *media* (“*prensa*”). First, *media* and *democracy* were related to the most negative and to the most positive sentiment, respectively. In addition, *authority* and *corruption* are words that are close to each other but present far away positions from *media* and *management*. The potential rationale supporting these relationships could be founded on the continuous criticism shown by the Spanish population concerning the decisions made by their government. In fact, the Spanish government has been repeatedly denounced over its management of the COVID-19 crisis. More concretely, the lack of foresight and the absence of official scientific experts supporting the decisions was the source of the majority of the complaints from the other political parties and the population. In addition, the shortage of protective material for health professionals, the purchase of defective coronavirus tests, and the distribution of thousands of faulty face masks to medical staff also created an overall sense of the government’s ineptitude.

In the case of Peru, the following words related to the *health* topic are presented in Figure 5b: *lockdown* (“*encierro*”); *isolation* (“*aislamiento*”); *depression* (“*depresión*”) *restriction* (“*restricción*”); *psychologist* (“*psicólogo*”); *mobility* (“*movilidad")*; *deceased* (“*fallecido*”); *confinement* (“*confinamiento*”); *COVID*; and *pandemic* (“*pandemia*”). Notice that *lockdown*, *isolation*, and *depression* are negative words that appear together. The size of the word *confinement* indicates that this is a constant source of discussion on social media, probably due to the fatigue induced by the lack of observable positive attitudes. Peru represents one of the countries with the highest COVID-19-related mortality rates in the world. The country is experiencing difficulties to accessing the vaccines and is also having their intensive care units completely overwhelmed. Due to these facts, regions are taking a long time overcome their assigned alert levels (currently categorised as high, very high, or extreme, with corresponding levels of restrictions in each case), which likewise leads to enforcing severe mobility, capacity and curfew restrictions, among others. The effects emerging from these circumstances could explain the negative emotions highlighted in Figure 5b.

In Mexico (see Figure 5c), the following words related to the *economy* topic presented: *business* (“*negocio*”); *company* (“*empresa*”); *debt* (“*deuda*”); *consequence* (“*consecuencia*”); *recovery* (“*recuperación*”); *rescue* (“*rescate*”); *economic* (“*económica*”); *bankruptcy* (“*quiebra*”); *aid* (“*ayudas*”); and *leadership* (“*liderazgo*”). In this case, the most positive (*company*) and negative (*debt*) words appeared close to each other, which could reflect the severity of the economic crisis caused by the pandemic. The same proximity is observed in the words *consequence*, *recovery*, and *rescue*, all of which hold a negative sentiment probably induced by the absence of the short-term expectation of seeing improvements in the economy. In fact, the highly open Mexican economy was heavily impacted by a severe reduction in the export demand. Additionally, it was also very affected by the decline in oil prices and the global market volatility. On 14 May 2020, the Mexican government announced plans to commence with the normalisation of economic activities, including a colour-based system (green–yellow–orange–red) to indicate the extent of activities allowed in the different states. Despite the coloured restrictions, cases and deaths began to rise again in December 2020, and in February 2021, they were forced back into declined due to the start of the second wave. The negative emotions shown in Figure 5c could be derived from these aforementioned past events.

Finally, in Chile, the following words related to the *employment* topic are presented in Figure 5d: *rent* (“*alquiler*”); *unemployment* (“*desempleo*”); *meeting* (“*reunión*”); *boredom* (“*aburrimiento*”); *lockdown* (“*encierro*”); *dismissal* (“*despido*”); *jobless* (“*paro*” or "*desempleo*”); *performance* (“*rendimiento*”); and *hunger* (“*hambre*”). Notice that the first word (*rent*) is not very related to the other words. The words *dismissal*, *performance*, and *unemployment* appear together in a clear reflection of the problems emerging from the difficulties that companies are facing to keep their staff on payroll. In fact, in March 2021, Chile remained under state of emergency and a daily nationwide curfew. The gradual plan designed by the government to reopen the economy and phase out the enforced quarantine measures presented some difficulties, since it was applied at the municipal level and depended on several criteria (reproduction rate of the virus, hospital bed occupancy, and the projected rate of regional active cases). This fact could lead to a possible reverse of the years of growth in the employment rate observed in the middle class in Chile. These circumstances could possibly be driving the negative emotions detected in Figure 5d.

**Figure 6:**
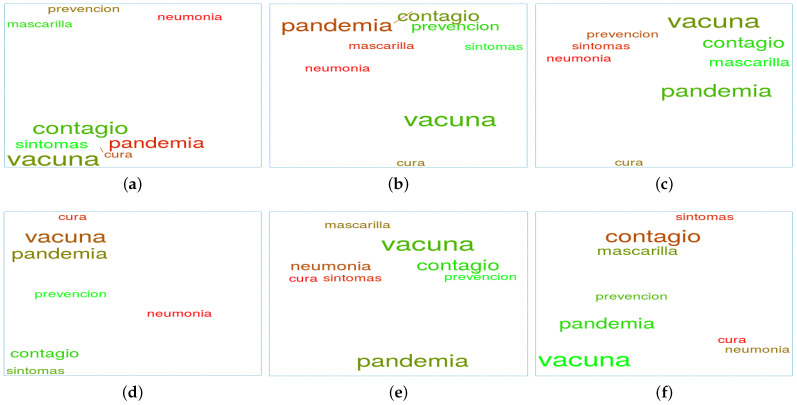

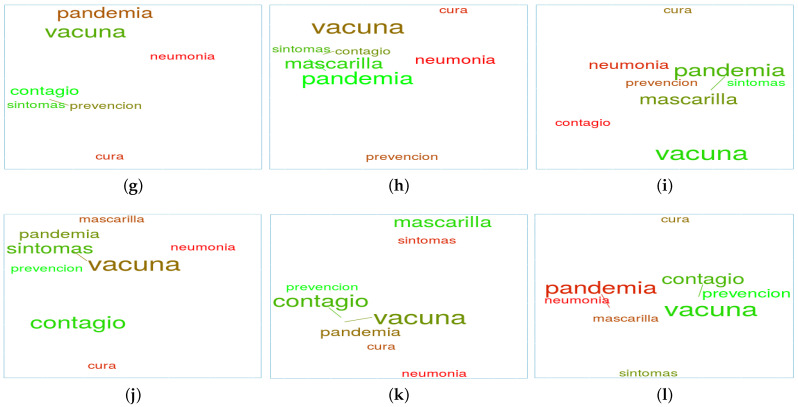
Sentiment evolution of vaccines topic in the different countries: (**a**) Chile, March 2020; (**b**) Chile, Sept. 2020; (**c**) Chile, March 2021; (**d**) Mexico, March 2020; (**e**) Mexico, Sept. 2020; (**f**) Mexico, March 2021; (**g**) Peru, March 2020 (**h**) Peru, Sept. 2020; (**i**) Peru, March 2021; (**j**) Spain, March 2020; (**k**) Spain, Sept. 2020; and (**l**) Spain, March 2021.

The previous analysis explored the behaviour of the different countries with a selection of relevant words for each topic. Nonetheless, it becomes possible to confer a different perspective when the same set of words is selected and word clouds are created on different dates to jointly study word positions and potential movements between dates for all the countries. As an exemplification of this strategy, Figure 6 presents the relationships among a set of words belonging to the topic *vaccines* in the different countries of interest at different moments of the pandemic (March 2020, September 2021, and March 2021). The words chosen are: *vaccine* (“*vacuna*”); *pandemic* (“*pandemia*”); *contagion* (“*contagio*”); *symptoms* (“*síntomas*”); *cure* (“*cura*”); *pneumonia* (“*neumonía*”); *face mask* (“*mascarilla*”); and *prevention* (“*prevención*”). Reasons to proceed with the aforementioned word selection are based on the impact and the number of occurrences during the period under review.

It is important to note that the same set of words is chosen for every country in such a way that a proper baseline is established and thus, comparisons can be performed. From these figures, several trends or behaviours can be observed.

For instance, in Chile, the word *pandemic* started with a negative sentiment (red colour) and evolved towards a positive perception (green colour) by March 2021. This could be explained by a growing hope regarding the end of the pandemic at the country level and the overall agreement (to a certain extent) on the positive appraisal for the crisis management exerted by the government. In addition, it is worth mentioning that the words *vaccine* and *cure* present considerable distance from one another for March 2021, as opposed to the initial proximity exhibited for March 2020. In line with this observation, the word *symptoms* similarly follows a separation process from the word *vaccine*, and it also reduces its frequency (i.e., size) over time. On the contrary, the words *prevention* and *face mask* closely surround the word *vaccine* for March 2021 (despite their considerable initial distance portrayed during March 2020). This new relationship could indicate that Twitter conversations discarded a potential short-term cure for COVID-19 and that they concentrated their efforts in discussing the prevention methods available to prevent further infections.

With regard to Mexico, it is interesting to note that words *vaccine*, *pandemic*, and *cure* are close to each other for March 2020 and then tend to separate over time. This quite likely reflects the initial situation with a lack of available information about COVID-19. Likewise, it may also represent the initial population concerns regarding the actual assessment of pandemic seriousness and the proper solution availability for it. The subsequent graphs allow inferring that much more meticulous conversations were taking place on Twitter from a pandemic perspective. In this regard, words such as *contagion*, *face mask*, and *symptoms* appear close to each other, demonstrating more awareness with respect to the pandemic. It is also relevant to note that the words *vaccine* and *pandemic* follow an ascending sentiment trend over time (colour turning light green). This serves as explicit evidence of the positive perception of the advances achieved in the fight against COVID-19.

In Peru, the word *cure* is always positioned far away from the rest of the words. This fact diverges from the rest of the countries, where the word *cure* is related to other words in at least one of the periods considered. As for the word sentiment values, some of them exhibit fluctuations over time, therefore missing a clear trend towards positive or negative value regions. This instability entails a true reflection of the apprehension and mistrust of the Peruvian population with respect to the unsuccessful courses of action taken by their government to manage the COVID-19 crisis (which some have reported to be the most severe amongst all Latin American countries). The words *vaccine*, *contagion*, and *pandemic* are clear instances of this behaviour.

As for Spain, it is relevant to note that the word *pandemic* evolved towards a very negative sentiment registered in March 2021, as well as being a word frequently found in tweets. This could be explained by the well-known Spanish population’s dissatisfaction regarding the absence of a clearly defined pro-active government strategy to deal with the COVID-19 pandemic, especially considering the extreme economic impact it has had on Spain, and the positive evolution observed in some European Union countries (France, Germany, and Portugal, among others). This tendency is also noticed in the sentiment associated with the word *face mask*. For this word, a negative trend is detected from September 2020 to March 2021, presumably due to the population’s disagreement on the use of face masks in open spaces when safe distance can be ensured. With respect to the potential word relationships, the graph depicted for March 2021 denotes a concern with all factors involved in the COVID-19 pandemic, whilst also being present during the previous months.

**Figure 7:**
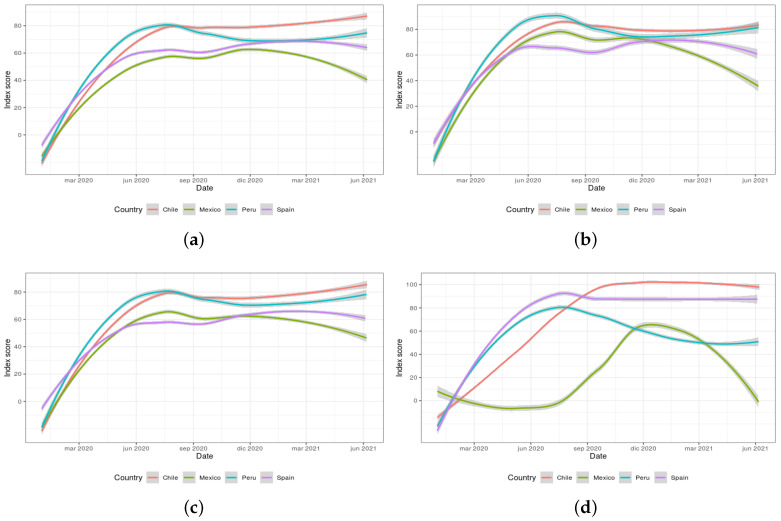
OxCGRT dataset global indexes for Chile, Mexico, Peru, and Spain: (**a**) overall government response index; (**b**) stringency index; (**c**) containment and health index; and (**d**) economic support index.

Figure 7a–d exhibit the scores of the four global indexes (overall government response, stringency, containment and health, and economic support) calculated by the OxCGRT dataset for the period from January 2020 to June 2021 for the selected countries. These indexes are calculated as an aggregation of several related individual policy response indicators. It is worth mentioning that the indexes do not provide assessment information on the enforcement of the policies or indicate a measure of the appropriateness or effectiveness of a particular government response. In addition, they do not capture the specific demographic or cultural features applicable to each country. On the contrary, the actual purpose of these indexes resides in providing a score to effectively allow cross-national comparisons of government interventions (the higher the score, the more severe the intervention).

In this regard, Figure 7a allows inferring that all governments implemented important responses at the beginning of the pandemic, with Chile being the more interventionist country over time in comparison with Mexico. As for Figure 7b, all countries show a similar level of stringency for December 2020 but evolve differently afterwards. Mexico becomes the country with the fastest relaxation of internal policies applied while Peru shows to be the country forced to strengthen them. In respect to Figure 7c, it is interesting to observe that Spain was forced to make their policies stricter to face the third wave which occurred around September 2020, just after a period in which the policies had been relaxed (probably too early). In contrast, Mexico followed a relaxation of the applied policies since that month, highlighting it as the most permissive of the selected countries. Finally, Figure 7d shows the consistency of Chile and Spain in their economic responses, clearly in contrast with those of Mexico and Peru.

The observation of the global indexes provided by the OxCGRT dataset provides some insights for further discussion of the word clouds formerly presented in Figure 5 and Figure 6. In Figure 5b, Peru shows negative sentiments towards the health topic-related words, which also shows alignment with the data presented in Figure 7c (as policies started to be enforced again around March 2021). Regarding Figure 5c, it is interesting to note the bad perception of the economy topic in Mexico in March 2021. This fact could be explained by the fluctuations in the enforcement of the economic policies taken by the Mexican government, as illustrated in Figure 7d. With respect to Figure 6a–c, the overall positive evolution of the perception of the words related to the vaccines topic in Chile could be linked to the stability and clear direction shown by the government to apply policies and measures in Figure 7a–c (as it can be inferred from the minor fluctuations displayed). Finally, with reference to Figure 6j–l, the negative trend detected related to the Spanish population’s dissatisfaction with regard to the management decisions taken by their government could be explained by the fluctuations shown in Figure 7a. These oscillations manifest a relevant inconsistency in the restrictions applied. In addition, an index peak can be found in March 2021, matching the negative trend detected in Figure 6l.

**Figure 8:**
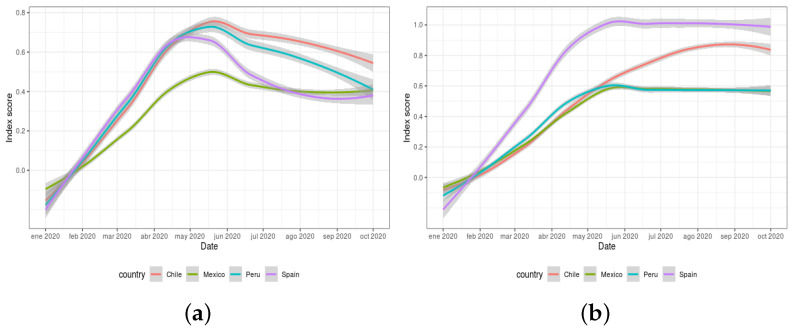
Response2covid19 dataset global indexes for Chile, Mexico, Peru, and Spain: (**a**) rigidity of public health index; and (**b**) economic intervention index.

A similar contextualisation exercise can be performed by using the two global indexes defined in the Response2covid19 dataset (rigidity of public health and economic intervention measures). These indexes are calculated by the aggregation of the responses collected for a total of 20 individual policy indicators. Figure 8a,b depict the evolution of both indexes during the period from 1 January 2020 to 1 October 2020. The values for these indexes range from 0 (no intervention) to 1 (all interventions are strict).

A comparison with the information provided in Figure 7 for the same period of time provides certain similarities (despite the fact that the individual policies from which these global indexes were obtained actually differ). In this respect, Figure 8a illustrates analogous information to that exhibited in Figure 7c, with Chile and Peru close in their respective rigidity of measures, and with Mexico enforcing less restrictions than the other countries. As for Figure 8b, it can be noticed that Spain implemented the most restrictive economic measures at the beginning of the pandemic, as also shown in Figure 7d.

More conclusions could be stated from the word clouds obtained, especially by means of a further contextualisation process with the specific scenarios drawn by each of the countries of interest. Furthermore, augmenting the input dataset could also procure a more accurate correlation between the word relationships observed and the real factors originating them.

## 5. Conclusions

This paper presented a study that covered the evolution of the general sentiment expressed on Twitter by its users during 13 months (from March 2020 to March 2021). These users were from four different countries where the dominant language is Spanish: Chile, Mexico, Peru, and Spain.

The textual information provided by Twitter was filtered by using manually selected keywords (selection oriented by a general literature review) related to the COVID-19 outbreak. These keywords were related to five different topics of interest. Then, the outcome was filtered again using the selected countries to ensure that only tweets with country information were used. This process generated an acceptable number of tweets which were manipulated through text mining techniques.

On the other hand, an enhanced version of the *EmoWeb* framework was developed to adapt the processing capabilities of textual information generated in social networks. This adaptation was the tool in charge of computing the emotional evolution. Thus, it took as input the lemmatised concepts extracted from tweets to learn the new sentiment values of words.

Regarding the experiments, they illustrate how the different situations related to the pandemic event (i.e., the multiple pandemic waves) affected the general sentiment of the individuals. The distinct measures considered by the selected countries were reflected in the emotional expressions of the users. Therefore, it can be stated that social networks are a good measure to follow the emotional state of different populations in response to traumatic events such as the COVID-19 outbreak.

In the future, it could be interesting to implement this solution using more complex systems based on deep neural networks such as BERT or similar ones. Notice that *EmoWeb* is based on a lexicon and uses simple concepts at the lexical level of the language. However, ML models are usually focused on distributional semantics which can be very interesting for lexicon-based approaches. Furthermore, it would be relevant to make comparisons between countries with very different anti-propagation measures or non-existent ones. In this study, the selected countries are sufficiently heterogeneous as they present differences between them; however, for instance, Brazil could provide more relevant information due to its extremely different strategy against the virus.

## Figures and Tables

**Table 1:** Vaccination policy—values description.

Value	Target Groups
0	No availability
1	Key workers, clinically vulnerable groups, elderly groups (just one)
2	Key workers, clinically vulnerable groups, elderly groups (just two)
3	Key workers, clinically vulnerable groups, elderly groups (all)
4	All three plus partial additional availability (select broad groups/ages)
5	Universal availability

**Table 2:** Vaccination policy—Oxford COVID-19 Government Response Tracker.

Country	1	2	3	4	5
Chile	24 December 2020	4 February 2021	1 March 2021	24 March 2021	Not started
Mexico	24 December 2020	15 February 2021	1 May 2021	Not started	Not started
Peru	6 February 2021	9 March 2021	28 March 2021	Not started	Not started
Spain	Skipped	27 December 2020	18 May 2020	Not started	Not started

**Table 3:** Number of tweets resulting from the data filtering processes by topic and country of interest.

Country	Government	Health	Economy	Employment	Vaccines	Total
Chile	709	584	2407	4131	6928	14,759
Mexico	1142	1785	4294	9552	15,940	32,713
Peru	605	222	911	1722	3456	6916
Spain	4713	3654	12,032	11,453	19,257	51,109

## Data Availability

Our study did not report any data or made any dataset publicly available. However, it made use of two public datasets: https://www.bsg.ox.ac.uk/research/research-projects/covid-19-government-response-tracker (accessed on 28 June 2021), https://response2covid19.org/ (accessed on 28 June 2021).
